# A Transformer-Based Capsule Network for 3D Part–Whole Relationship Learning

**DOI:** 10.3390/e24050678

**Published:** 2022-05-11

**Authors:** Yu Chen, Jieyu Zhao, Qilu Qiu

**Affiliations:** Faculty of Electrical Engineering and Computer Science, Ningbo University, Ningbo 315211, China; 1801082071@nbu.edu.cn (Y.C.); 1911082062@nbu.edu.cn (Q.Q.)

**Keywords:** 3D shape transformer, local-to-global cognition, shape-Transformer-based capsule, deformable 3D object

## Abstract

Learning the relationship between the part and whole of an object, such as humans recognizing objects, is a challenging task. In this paper, we specifically design a novel neural network to explore the local-to-global cognition of 3D models and the aggregation of structural contextual features in 3D space, inspired by the recent success of Transformer in natural language processing (NLP) and impressive strides in image analysis tasks such as image classification and object detection. We build a 3D shape Transformer based on local shape representation, which provides relation learning between local patches on 3D mesh models. Similar to token (word) states in NLP, we propose local shape tokens to encode local geometric information. On this basis, we design a shape-Transformer-based capsule routing algorithm. By applying an iterative capsule routing algorithm, local shape information can be further aggregated into high-level capsules containing deeper contextual information so as to realize the cognition from the local to the whole. We performed classification tasks on the deformable 3D object data sets SHREC10 and SHREC15 and the large data set ModelNet40, and obtained profound results, which shows that our model has excellent performance in complex 3D model recognition and big data feature learning.

## 1. Introduction

How to make neural networks understand images like humans is very difficult, because the human visual system is very complicated. There is strong psychological evidence that people interpret visual scenes as parts of the overall hierarchy [1]. So if we want the neural network to understand images like humans, we need to figure out how the neural network represents the part–whole structure and model the spatial relationship between the part and the whole. Similarly, in the feature learning of a three-dimensional model, since the model itself has quite a wealth of space and shape information, it is more important to be able to learn the relationship between its local shape and the whole for the model.

Many previous works are based on scalar convolutional neural networks (CNNs) to learn local feature representations for 3D models [2,3,4]. However, due to the limitation of the convolutional receptive field, the convolutional network can only construct feature relationships in local regions, and there is no attention mechanism to capture different feature relationships according to different tasks. The above issues seem to be addressed to varying degrees, as new Transformer architectures have been successfully used for vision-based tasks [5]. These Transformer architectures do not have the structural induction bias provided by convolution to the local spatial structure. Instead, they are completely based on flexible attention distribution. This mechanism enables us to quickly establish the relationship between each local patch. Therefore, we hope to extend the Transformer to the three-dimensional domain and build the associations between local shapes on this basis. However, it is difficult to design a neural network for a mesh model, especially to add information, such as direction, position, and the relationship between the part and the whole in the three-dimensional model. The obvious difficulty is that the mesh is usually composed of a series of irregularly distributed vertices and edges. Although the topological information can clearly express the geometric shape of the surface and can reliably describe the complex surface, it is difficult to directly apply the traditional deep learning framework to this data format. So we not only have to solve the problem of irregular distribution of vertices in 3D models, but also think about how to provide geometrically interpretable tokens as input to the shape Transformer.

In addition, the representation of each node in the CNNs will be treated as multiple individual scalar features instead of a vector, which may not be enough to effectively preserve the spatial and shape information of the three-dimensional model. On the other hand, CNNs lose a lot of information in the pooling layer. They tend to ignore the spatial arrangement in the data, and therefore do not respect the integral part–whole relationship that is essential for explaining and describing 3D shapes. This is also the difficulty we face when establishing the relationship between local shapes.

Some recent studies have tried to introduce vector network to save relevant position and structure information to solve this problem [6,7]. For example, in the recent research hotspot of capsule networks [8], detailed pose information (e.g., precise target position, rotation, thickness, tilt, and size) is preserved throughout the network via sequestered vectors, rather than being lost and then recovered. The capsules are organized in multiple layers to realize the coding of the visual scene. The low-level capsules can be used to represent low-level visual entities (for example, edges or object parts), while the high-level capsules can represent the entire objects, and the connections between the low-level and high-level capsules are implemented using routing algorithms. With such explicitly grouped visual representations, structural information will be more reasonably preserved compared to CNNs.

Inspired by the above work, we propose a vector-type mesh trans-capsule neural network based on shape representation. Our mesh Transformer features are treated as the underlying 3D local shape description, which is then combined into multiple parts (larger local surfaces), and finally these parts are combined into the entire object in a learning manner. Specifically, in order to obtain appropriate Transformer features, we design a mesh Transformer as a novel 3D local shape-based attention mechanism. This mechanism can find the similarity between the local surface Query (Q) and the shape template Key (K), and form a feature vector about the shape encoded with different weights. Subsequently, we designed a multi-head attention mechanism for these shape features to form different sets of multilayer perceptrons. The multi-head attention mechanism can provide different subspace encoding representation information for the output of the attention layer so as to more comprehensively describe the relationship between local shape information and encapsulate it in a capsule. We organize the capsules in a hierarchical dynamic routing way to learn the mapping from part to the whole, and finally realize the encoding of the entire object.

In summary, the main contributions of our work are summarized as follows:

- 3D shape Transformer. We propose a novel self-attention calculation method based on local shape representation. It allows a mechanism similar to the standard 1D self-attention, taking the local shape of the mesh model surface as a token, and designing a matching similarity measure for it. Thus, the well-known 1D Transformer suitable for NLP can be adapted to 3D mesh tasks.
- Multi-head shape attention layer. We propose a multi-head shape attention mechanism to form multiple subspaces, allowing the model to pay attention to different aspects of information. This expands the possibility of combination between the underlying local shapes, and makes the local combination information learned by the model more accurate.
- Vector representation. Based on the 3D mesh data, we propose a new primary capsule construction method to improve the performance of the capsule network.
- 3D vector-type network. We construct a novel vector-type mesh trans-capsule neural network and apply it to the recognition of three-dimensional deformable models. Experiments show that compared with other methods, our network can respect the geometric characteristics of the model itself, and has a better classification effect and learning ability.

## 2. Related Work

### 2.1. Transformer for 2D Vision

Inspired by the success of the self-attention layer and Transformer architecture in the NLP field, some works try to extend Transformer to the field of image recognition. In vision applications, CNNs were once considered the fundamental component [9,10], but nowadays Transformer is showing that it is a viable alternative to CNN. Dosovitskiy et al. [5] proposed Vision Transformer (ViT), the first attempt to apply the standard pure Transformer directly to the image with as little modification as possible. To this end, they divided the image into small blocks and converted these blocks into linear embedding sequences as input to Transformer. They proved that the dependence on CNN is not necessary. In the image recognition task, applying the pure Transformer model to the sequence of image patches can also perform well. He et al. [11] proposed a novel Transformer-based framework, TransFG, to apply Transformer to fine classification tasks. They integrated all the original attention weights of Transformer into the attention map to guide the network to effectively and accurately select distinctive image blocks and calculate the relationship between them. Liu et al. [12] proposed a new visual Transformer module called Swin Transformer, which can be used as the general backbone of computer vision. This method introduces CNN’s local area and hierarchical feature ideas into Transformer. Of course, it is not only suitable for basic image classification problems, but Transformers are also used to solve various other computer vision problems, such as object detection [13,14], semantic segmentation and video understanding.

Inspired by the local patch structure used in ViT and the basic semantic information in language words, we propose a local patch representation method based on the topological connection of the 3D mesh model surface, and design a novel shape embedding representation that makes this local patches can visually represent shape information. Therefore, we consider the local shape fragments as the smallest unit, through the calculation of the similarity of the local shapes and the introduction of the multi-head shape attention mechanism to realize the learning of the combination weights between the local shapes.

### 2.2. Transformer for 3D Vision

Unlike 2D images, 3D data are disordered and unstructured, making it challenging to design neural networks to process them. Hermosilla et al. [15] proposed the Monte Carlo convolution neural network (MCCNN) to describe convolution as a Monte Carlo integration problem. This method guarantees that the potential nonuniform sample distribution function is fully considered from the perspective of Monte Carlo, thus cleverly avoiding the problem of directly processing disordered structure data. Li et al. [16] proposed learning the *X* transformation from the input points to simultaneously weight the input features associated with the points and rearrange them into a potentially implicit canonical order. This method applies the element multiplication and sum operation of the typical convolution operator to the *X* transformation feature. MeshNet [17] uses the extracted information from the triangular mesh data as features and uses the output of the feature space description module and the structure description module as the input of mesh convolution to build a deep learning network that can directly process the mesh model. Furthermore, Biasotti et al. [18] analyzed and evaluated state-of-the-art retrieval and classification algorithms dealing with an emerging field, namely textured 3D objects. This not only provides a reference for researchers on shape descriptors, but also provides a deep learning direction on color features. Rodolà et al. [19] evaluated the performance of 15 retrieval algorithms based on the 3D deformable mesh model SHREC17.

With the breakthrough progress of Transformer in the field of NLP, there have been many recent works on extending Transformer to 3D data. Compared with the original self-attention module in Transformer, Wang et al. [20] proposed an improved self-attention using implicit Laplacian and normalization. Zhao et al. [21] designed a point Transformer layer for point cloud processing and designed a hierarchical network structure. Lin et al. [22] used a graph convolution named Graphormer to improve Transformer. This approach only adds a graph convolution module to the attention module to fuse local information, which forms global and local friendly structures, thereby realizing 3D human pose estimation and mesh reconstruction based on a single image. However, these methods do not have careful consideration for the design of the calculation of the 3D self-attention mechanism; therefore, local features cannot be captured in detail. Moreover, the embedding representation of the input is not as interpretable as the token in NLP. It is important to note that a Transformer network suitable for 3D mesh models has not yet been proposed. However, the 3D mesh Transformer model that we propose can overcome the limitations described above.

### 2.3. Vector Networks for 3D Vision

Although the convolutional network has a good effect in the field of three-dimensional model recognition, due to the existence of the pooling layer, the structure and position information will be lost in the convolution process. To solve this problem, many studies have introduced vector representations into neural networks to maintain the rotation and translation of features [7,23,24,25].

Hinton et al. [24] first proposed the idea of a capsule network. They proposed a vector encapsulation method called “Capsule” and designed a method for dynamic routing through a protocol, thus starting the latest work on this topic. Since then, many studies have emerged by combining the routing with several well-known concepts, such as equal variance clustering [26], Kullback–Leibler divergence regularization [27] and expectation–maximum algorithms [28]. They have even been extended to the field of generation, such as the combination of autoencoders [6] and generative adversarial networks [29].

However, up until now, the application of the capsule concept in the 3D domain has been rather uncharted territory. In recent years, a small amount of groundbreaking work has attempted to apply capsule networks to 3D data. Zhao et al. [7] proposed a 3D point capsule network that uses capsules to learn a specific part of an object, and confirms that the capsule representation has the possibility of extracting semantics from 3D point clouds. However, this model only learns the mapping of three-dimensional points to component-level capsules, and does not learn the combination of component-level capsules. In addition, this method does not decompose features into pose and feature components separately, which reduces its geometric interpretability. Recently, quaternion equivariant capsule networks (QECN) [30] were proposed to specifically learn the pose representation of objects. The model is built as a multi-level structure based on local representations, where each local representation is modeled as a quadruple. However, the model is learned through a supervised training method of category labels and relative positions. Based on the QECN, the geometric capsule autoencoder (GCA) [25] uses an unsupervised method to learn the entity representation from the 3D point cloud. In order to allow each capsule to learn the posture and feature vector at the same time, the model decomposes the representation of the posture and feature into a translation vector and a quaternion vector tuple. Through the joint learning of posture and features, the feature representation of the entire object is obtained.

Although the above methods have made pioneering contributions in the field of 3D capsules, they do not apply the concept of capsules to 3D mesh models, much less consider the combination between component capsules in a geometric sense using the local shape information and topology of the mesh model.

## 3. Methods

### 3.1. 3D Mesh Trans-Capsule Networks Architecture

**Figure 1:**
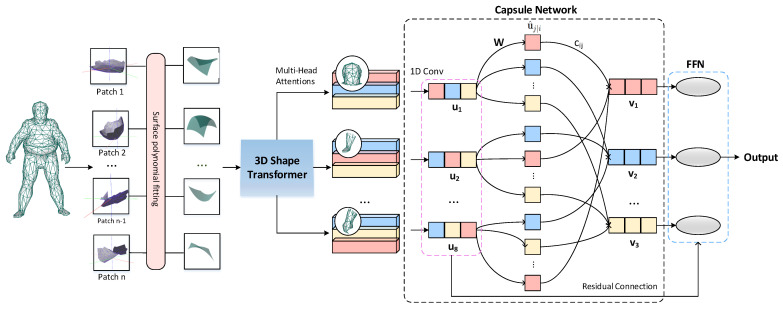
3D mesh trans-capsule network architecture. Our network accepts NX3 mesh data as input. By sliding the window on the surface of the mesh model to obtain patches similar to the two-dimensional image Transformer, and use a polynomial function to fit the three-dimensional data in each partial window. We use the typical surface shape obtained by unsupervised learning as a simple expression of Q and K, and perform the attention mechanism calculation on the shape data in the local window to obtain the features. These features are then constructed as primary capsules, and we then learn combinations between features through iterations of routing.

We describe the overall structure of the proposed 3D mesh trans-capsule networks in this section, and its structure is shown in Figure 1. Among them, the 3D shape transformer is used to obtain the feature representation of the local shape and build the relationship between each local patch. Then, multiple subspaces are formed through the multi-head shape attention mechanism to expand the possibility of combining the underlying local shapes, and realize the mapping encoding of the mesh model from local shape fragments to large shape parts. Finally, we introduce a vector network to learn the mapping from large parts to whole objects. Throughout the pipeline, we use vector representations to build shape encoders that fully contain the spatial structure information of the 3D model.

The input of the network is an N×d mesh data, where we set *N* to be the number of points of the model in the data set and *d* to be the feature dimension of the input. In many representative studies, N=1024 or 2048 is usually set. Due to the experimental environment, we set N=700 in our paper. Nevertheless, our proposed model still has excellent performance on the classification task of 3D non-rigid models.

For a typical 3D data set *d* = 3, but in order to reflect the connection information of the mesh surface, we add the approximate geodesic distance so that *d* = 4. Similar to two-dimensional vision Transformer, we use the surface mesh transformer network proposed in this paper to extract local surface shape features. We use the local shape patches sampled on the model surface as the input set, namely Q. In theory, Q and K come from the same input. In order to reduce the computational cost, we perform unsupervised clustering on the surface of the local window to obtain a typical shape patch, which is regarded as K. In fact, the essence of Q and K is the same; we set the number of typical shape patches to *C*. We realize the attention calculation by measuring the similarity between the local surface window and the typical local shape. Then, we introduce a multi-head shape attention mechanism to expand the possibility of combination between the underlying local shapes and make the local combination information learned by the model more accurate. In order to diversify the learning as suggested by capsule networks, we feed these feature maps which describe shape information into a point-wise multi-layer perceptron (C−1024) with different weights, each with a distinct summary of the input shape with diversified attention. We then maxpool their responses to obtain a global latent representation. These descriptors are then concatenated into a set of vectors named primary capsules. We then use the dynamic routing to embed the primary capsules into higher level latent capsules. Each capsule is independent and can be considered as a cluster centroid (codeword) of the primary capsules.

### 3.2. 3D Shape Transformer

The goal of the entire 3D trans-capsule network design is to achieve the mapping of local shapes to the overall model. We choose the Transformer model to realize the feature learning of the local basic shape. Inspired by the local patch structure used by ViT and the basic semantic information of words in NLP, we propose a local shape token representation. This representation can describe the shape of local patches on the surface of the 3D mesh model, thereby capturing local geometric features and obtaining semantic information. This section mainly introduces the 3D local shape token representation and its feature learning module 3D shape Transformer to show how to implicitly encode the geometric and structural information of the local area in the mesh space.

#### 3.2.1. The Definition of Local Shape Token

In this part, we mainly introduce the definition of local shape tokens and the similarity measurement between local shapes. Similar to the two-dimensional ViT, we design a sliding window on the surface of the grid model to obtain a local surface shape with a fixed number of points and a variable grid size, and then regard it as an input element with a similar position as the word in NLP, namely the shape token. Then, a specially designed 3D patch-based Transformer module is used to extract local shape features of the mesh model.

Given a 3D mesh model $M^{S}$, we define the local mesh window of the model as follows: Taking the vertex $v_{i}$ in the model as the center of the local window, a breadth-first search is used to obtain the first K−1 neighboring vertices. We regard the mesh surface formed by the selected vertices and the edges between the vertices as the local mesh window.

It is worth mentioning that our proposed local shape representation method is very suitable for the feature description of non-rigid models because under normal circumstances, the surface shape distribution of the 3D model changes very slightly during deformation.

We use the high-order polynomial equation $F\left(v^{c}|\theta \right)=0$ to describe its shape in the local coordinate system, where $F\left(v^{c}|\theta \right)$ is the continuous function of the local surface, and $v^{c}$ is the relative coordinate of the vertex in the window, and θ is defined as a parameter of the function. During the experiment, it is found that if the window size *K* is set to be too small, the surface shape tends to be uniform and difficult to distinguish, and when *K* becomes larger, the inner surface of the window is more complicated. Therefore, the approximate geodesic distance d of the vertex relative to the center of the window is used in the fitting for a better description. Finally, we take $v^{c}=\left(x,y,z,d\right)$ as the window surface fitting function.

(1) $$\begin{array}{cc} F\left(v^{c}|\theta \right) & =z-\left(\theta_{0}+\theta_{1}x+\theta_{2}y+\theta_{3}d+\theta_{4}x^{2}\right.\left.+\theta_{5}y^{2}+\theta_{6}d^{2}+\theta_{7}xy+\theta_{8}xd+\theta_{9}yd\right) \end{array}$$

where the parameter $\theta =\left(\theta_{0},\theta_{l},\cdots ,\theta_{9}\right)$ is calculated using the generalized least squares method (GLS).

Given two different vertices $v_{R}$ and $v_{T}$, the corresponding local surface functions $M_{R}$ and $M_{T}$ centered on $v_{R}$ and $v_{T}$ are expressed as $F_{R}\left(v^{c}|\theta_{R}\right)$ and $F_{T}\left(v^{c}|\theta_{T}\right)$) respectively. We define the one-way shape difference distance from $M_{R}$ to $M_{T}$ as,

(2) $$\mathrm{Diff}\left(M_{R},M_{T}\right)=\sum_{v_{r}\in M_{R}}F_{T}^{2}\left(v_{r}^{c}|\theta_{T}\right)$$

where $v_{r}$ is an arbitrary vertex in the surface $M_{R}$. In order to reduce the error caused by fitting, we define the two-way shape difference distance between two local surfaces as,

(3) $$\mathrm{Diff}<M_{R},M_{T}>=\frac{1}{2}\left[\mathrm{Diff}\left(M_{R},M_{T}\right)+\mathrm{Diff}\left(M_{T},M_{R}\right)\right]$$

#### 3.2.2. The Definition of Typical Local Shape Token

In the last section, we surfaced the local patch to form a shape token, and on this basis, we calculate the self-attention mechanism. However, the self-attention mechanism needs to calculate the similarity between tokens, which will cause a larger amount of calculation, even more in three-dimensional data. In order to reduce the computational burden, we consider replacing one of the query and key sequences with a typical shape token. The typical shape token is essentially a representation of the original local shape, so this is feasible. In this section, we introduce how to obtain a typical shape token.

Our typical local shape token method is inspired by the feature bag model. The bag-of-features model is modeled after the bag-of-words method in the text retrieval field. Each image is described as an unordered set of local patches/keypoint features. Use a clustering algorithm (such as *K*-means) to cluster local features. Each cluster center is regarded as a visual word in the vocabulary, which is equivalent to a word in a text search. The visual vocabulary is represented by the code word formed by the corresponding feature of the cluster center (can be regarded as a feature quantization process). In our method, the local window is moved on the mesh model, the model is divided into a series of mesh fragments, and a polynomial function is used to fit the shape of the fragments to form shape patches. After modeling the feature packet model, we can describe each mesh object as a disordered collection of these shape blocks. Clustering these local shape functions by clustering method, each cluster center can be regarded as a typical local shape. These typical local shapes can form a set of shapes (similar to a visual dictionary). In addition, in our method, these typical local shapes can be regarded as shape tokens, so that the input of each patch of the model can be described by shape tokens to form a local shape embedding representation in a uniform spatial dimension.

The similarity matrix is the distance measure between any two shape complements in the local shape set. The elements in the similarity matrix are constructed by the following formula:

(4) $$w_{ij}=\exp\left(\mathrm{Diff}<M_{R},M_{T}>\right)$$

where $w_{ij}$ is the element in the similarity matrix W. On the basis of building this similarity matrix, we use spectral clustering to obtain typical shape tokens.

**Figure 2:**
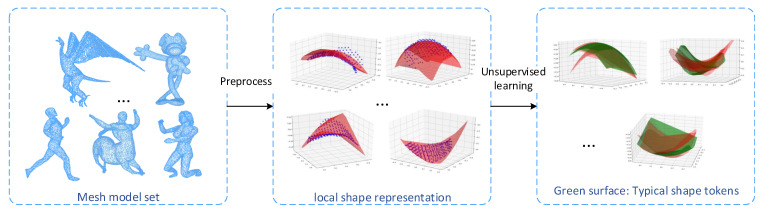
Obtain typical shape tokens through unsupervised learning. In the experiment, one of the models is randomly selected from each type of model and 10% of the vertices are taken as the center of the local shape. After that, polynomial fitting is performed on the local shape and unsupervised clustering of the surface set. The finally obtained cluster center is the typical shape token after polynomial fitting.

After obtaining the results of the local mesh shape clustering, the clustering center, as the “mean” of the same category mesh shape, is difficult to represent by a specific surface. Therefore, this method uses the polynomial function $F^{*}$ as the shape representation of the clustering center $M^{*}$. On the basis of the above clustering results, for each typical mesh shape, $S_{n}$, its class center is $M_{n}^{*}$, the shape representation is $F_{n}^{*}$, and $S_{n}$-class local shapes are used as the fitting data. Then, the parameter $\theta_{n}$ can be solved by using the least squares method, and the parameter $\sigma_{n}^{2}$ is calculated according to the following formula:

(5) $$\begin{array}{ccc} \sigma_{n}^{2}=\frac{1}{Num_{n}-1}\sum_{i}^{Num_{n}}Diff\left(M_{X_{i}},M_{n}^{*}\right) & \; & M_{X_{i}}\in S_{n} \end{array}$$

where $Num_{n}$ represents the number of surfaces classified as shape type $S_{n}$ in the clustering result. The detailed calculation method of the typical shape token is shown in Figure 2.

#### 3.2.3. 3D Shape Transformer

The first step in calculating self attention is to create three vectors from the input vector of each encoder. For each token, we create a query vector, a key vector and a value vector. These vectors are generated by multiplying the word embeddings by the three training matrices created during the training process. The second step is to score the corresponding words by using the dot product of the query vector and the key vector, and the resulting score set is the attention weight. Then multiply each value vector by the score after softmax. The actual meaning is to reduce the attention to irrelevant words while keeping the attention degree of the current word unchanged. Finally, the attention feature is defined as the weighted sum of all value vectors with attention weights. Obviously, the output attention feature of each word is related to all input features, so the global context can be learned.

**Figure 3:**
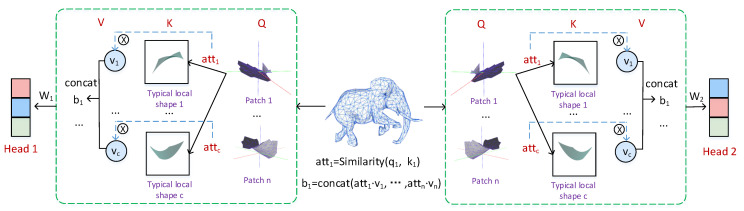
Shape attention. For a given mesh model, we first split the input 3D mesh object into patches through topological connection information. Each patch is regarded as a “shape token”, denoted as Q. A typical local shape token is a representative shape formed by unsupervised clustering learning of shape tokens to reduce the computational burden. In essence, it still represents the original patch, denoted as k. Then use the similarity between the shapes to calculate the attention weight. Finally, the weight and the corresponding value vector are multiplied to obtain the output of the shape attention.

In the design of the shape Transformer in this article, we respect the standard self-attention mechanism to the greatest extent [5]. We also designed $Q_{mesh},K_{mesh},V_{mesh}$ sequences in our method. Essentially, $Q_{mesh},K_{mesh},V_{mesh}$ should also represent the shape token as input. However, in order to reduce the space and time complexity, we define $Q_{i}\in Q_{mesh}$ as the initial local patch (ie shape token), $K_{i}\in K_{mesh}$ as the simplified local patch (i.e., typical shape token), and $V_{i}\in V_{mesh}$ as the feature value of the local patch. For the 3D mesh model without texture, we set the value of V to 1. The specific shape attention mechanism can refer to Figure 3.

We first designed a similarity measurement mechanism for shape tokens. Unlike VIT, which uses dot products to calculate the similarity between feature vectors, we specifically designed the similarity att between shapes. Assume that the distribution of this type of local shape in the 3D mesh model data set obeys the Gaussian distribution, and its distribution variance is σ. Then, in a given three-dimensional mesh model, the probability that the shape of the local shape token $q_{i}$ is similar to this type of typical local shape token $k_{i}$ is

(6) $$\begin{array}{cc} att_{i} & =Similarity\left(q_{i},k_{i}\right)=P\left(q_{i}|\theta_{k_{i}},\sigma \right)=\frac{1}{\sqrt{2\pi \sigma^{2}}}\exp\left(-\frac{\sum_{v_{q}\in q_{i}}F_{k_{i}}^{2}\left(v_{q}|\theta_{k_{i}}\right)}{2\sigma^{2}}\right) \end{array}$$

In order to make the attention weight more stable, finally in the experiment, we take the logarithm of the above formula to represent $att_{i}$:

(7) $$\begin{array}{c} att_{i}=\ln P\left(q_{i}|\theta_{k_{i}},\sigma \right) \end{array}$$

Finally we use the regular $F_{out}=S_{att_{i}}\cdot V$ to obtain the output. In our experiment, for a three-dimensional model containing texture features, *V* represents its feature value, otherwise *V* is set to 1.

In summary, the calculation process of the 3D shape Transformer we designed is shown in Figure 3. The local shape patch of the original data can be regarded as *Q*; the typical shape token with the same geometric properties as *Q* can be regarded as *K*; and the feature value of the local shape is *V*. The above calculation process becomes a complete attention calculation, that is, the output value of the local shape patch1 is $b_{1}=concat\left(att_{1}\cdot v_{1},att_{2}\cdot v_{2},\;\cdots ,\;att_{n}\cdot v_{n}\right)$, where att=Similarity(q,k). It is worth noting that our output *b* is no longer a value, but a set of vectors based on shape description.

### 3.3. ShapesToShapes and ShapesToObject

According to the traditional method, this paper can perform feature extraction by constructing a multi-level mesh Transformer, and the obtained features can be directly connected to the classifier for classification. But there are still some problems here, that is, the spatial combination relationship between the underlying shape features is not considered. Since only the relationship between the local shape tokens is paid attention to in the attention mechanism, and the neurons in the upper layer are passed as scalars to the neurons in the lower layer (the scalar has no direction and cannot represent the positional relationship between the upper layer feature and the lower layer feature). So in our network, we treat the local surface shape as the lowest-level token representation and design a special mesh attention mechanism for them. In order to learn the combination and geometric positional relationship between the underlying shapes, we introduced vector-type hidden units. This can not only express the 3D model with the intensity of the characteristic response, but also characterize the geometric structure information of the 3D model.

In ShapesToShapes, we introduce a multi-head shape attention layer to ensure the diversity of combinations between local shapes, and use a fully connected layer to realize the mapping between local shapes and larger parts. In the subsequent ShapesToObject, the dynamic routing mechanism is used to update the weights from one layer to the next between each part of the shape capsule, so as to propagate the attributes captured by the node capsule to the appropriate object capsule, and the mapping can achieve a larger part to the whole object. Therefore, each model is modeled as multiple partial capsules, which are then modeled as object capsules.

#### 3.3.1. Multi-Head Shape Attention Layer

On this basis, we propose a multi-head shape attention mechanism, as shown in Figure 3, to form multiple subspaces, allowing the model to pay attention to different aspects of information. This expands the possibility of combination between the underlying local shapes, and makes the local combination information learned by the model more accurate.

Different from the conventional multi-head attention mechanism, we perform a separate feature mapping for each subspace to maintain the diversity of relationships between its local shapes, thus laying a good foundation for the formation of the subsequent primary capsule layer. We think this is one of the reasons why the network of this article is effective.

#### 3.3.2. Routing-by-Agreement

We encapsulate the shape feature information extracted by the 3D ShapeTransformer into a primary capsule, and use a multi-layer routing-by-agreement to continuously map the partial shape to the overall model, thereby constructing the relationship between the part and the whole.

The reason why the dynamic routing algorithm is used to construct the local-to-holistic mapping is because in the capsule network, the lower-level features (hands, eyes, mouth, etc.) will only be transmitted to the matching upper-level. If the bottom features include features similar to eyes or mouth, it will be transferred to the upper layer of the “face”; if the bottom features include features similar to fingers, palms, etc., it will be transferred to the upper layer of the “hand”. So we imagine that in the representation of the three-dimensional model, we can also learn the combination of local shapes and the local-to-global mapping.

On the other hand we implement the dynamic routing algorithm for local parts to whole object mapping, i.e., PartsToObject part, and the dynamic routing algorithm is essentially a clustering method, which is in line with our idea of clustering from small parts to large objects.

In the mechanism of dynamic routing, the capsules are not directly connected to each other as in the case of full connectivity, but the output of the primary capsules generates a prediction vector, which is later weighted and summed with the corresponding coupling coefficients. The coupling coefficient $c_{ij}$ is determined by the softmax function, while at the beginning they initialize $y_{ij}$ equal to zero. At that time, it does not show the relationship between the capsule of the previous layer and the capsule of the next layer. The latest $y_{ij}$ is determined by the dot product of the previous $y_{ij}$ and the output $u_{ij}$ of the higher capsule. Finally, according to the squashing function, the output of the capsule is obtained. Finally, according to the squashing function, the output of the capsule is obtained.

Capsule networks allow multiple classifications to exist simultaneously, so the traditional cross-entropy loss function can no longer be used, but interval loss is used as the loss function, and the interval loss formula is shown below,

(8) $$\begin{array}{cc} L_{k} & =T_{k}\max{\left(0,m^{+}-∥u_{k}∥\right)}^{2}+\lambda \left(1-T_{k}\right)\max{\left(0,∥u_{k}∥-m^{-}\right)}^{2} \end{array}$$

where $L_{k}$ is the calculated interval loss; $T_{k}$ is the existence value of the *k*th classification, which is taken as 1 if it exists and 0 otherwise; $m^{+}$, $m^{-}$ and λ are taken as 0.9, 0.1 and 0.5, respectively.

## 4. Results

To verify the effectiveness of the method in this paper, we conducted experiments on the standard three-dimensional deformable mesh model data sets SHREC10, SHREC15 and the large dataset, Manifold40.

### 4.1. SHREC10, SHREC15 and Manifold40

The SHREC10 dataset includes 200 non-rigid 3D mesh models in 10 categories. There are 20 models in each category and the same models have rigid body transformation and non-rigid body transformation. In addition, in this dataset, the grid size of each 3D model is relatively uniform. When training the network, randomly select 14 three-dimensional models in each category as training samples, and the rest as test samples.

In order to further verify the effect of this method in more types of 3D model data sets, experiments are carried out using the SHREC15 data set. The SHREC15 data set includes 1200 three-dimensional mesh models in 50 categories, each with 24 models, and each model has rigid body transformation and non-rigid body transformation. When training the network, randomly select 17 three-dimensional models from each category as training samples, and the rest as test samples.

ModelNet40 [31], containing 12,311 shapes in 40 categories, is a widely used 3D geometry learning benchmark. However, most of the 3D shapes in ModelNet40 are not watertight or 2-manifold, which will affect the selection and fitting of local shapes. Therefore, we refer to article [32], which reconstructs the shapes in ModelNet40 and constructs the corresponding Manifold40 data set, where all shapes are closed manifolds.

### 4.2. Shape Classification

The classification task is often considered a litmus test for evaluating a network because a network that shows strong performance on the classification task can often be tuned to achieve strong performance on other tasks. The purpose of shape classification is to classify shapes into different classes. We evaluated the performance of our 3D mesh trans-capsule network on shape classification tasks on two non-rigid model data sets SHREC2010 and SHREC2015, and a large data set ModelNet40. Classification accuracy is measured using 80% training samples. These samples are randomly selected from each class as training samples, and accordingly, the remaining shapes in each class are considered as test samples. Our network achieves a correct classification rate of 100 on the SHEREC dataset at this ratio. Therefore, in order to further verify the capabilities of our proposed model, we will explore the impact of training and test samples at different ratios on the classification results in subsequent experiments.

We show the classification results on the data sets SHREC10 and SHREC15 and Manifold40 in Table 1, Table 2 and Table 3. We compared several methods aimed at using this data in different representations and using different core operators. Compared with other methods, our method achieves a classification accuracy of 100 on the SHEREC dataset. We believe that the higher performance of the 3D mesh trans-capsule network comes from the interpretability of the built model on the one hand. The model is designed based on imitating human vision, not just the stacking of models. Another aspect may come from the compactness of the structure. Due to its compactness, the training of the classifier is easy to converge, especially when the number of training samples is limited. On Manifold40, the data set is more challenging due to reconstruction errors and simplified distortions, and the accuracy of all other test methods is reduced. The 3D mesh trans-capsule network is once again superior to all grid-based methods on Manifold40.

### 4.3. Analysis of Parameters

**Figure 4:**
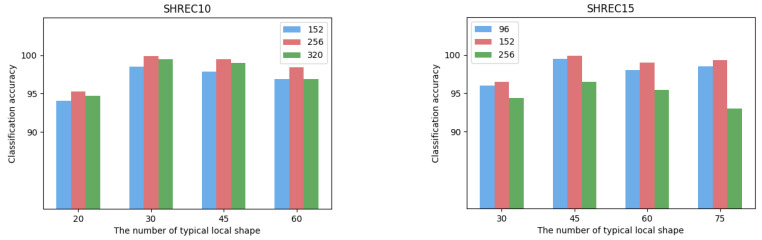
The effects of local size and number of typical local shapes on the classification accuracy on datasets of SHREC10 and SHREC15, respectively.

The size of the local shape is defined as the size of the local area centered on each vertex of the 3D mesh model, and its value will affect the feature extraction of the object. Based on the mesh densities of different datasets, we set the size of local shapes on the SHREC10 dataset to be 152, 256, 320 for ablation analysis. The sizes of local shapes on the SHREC15 dataset are set to 96, 152, 256. The comparison of the classification results obtained under different sizes of parts is shown in Figure 4. It can be seen from the figure that in the dataset SHREC10, the local shape size value of 256 has the best experimental performance. In the dataset SHREC15, the local shape size value of 152 has the best experimental performance. We believe that too small local shapes are difficult to describe more complex surface changes, and too large local shapes will lead to overly complex shape descriptions, resulting in learning difficulties. In addition, the grid density results in different local shape sizes in different datasets.

The typical local shape is a representation of the set of surface shapes in the entire dataset, and is proposed as a way to optimize the computational efficiency of the Transformer. We also compare and analyze the classification accuracy under different numbers of typical local shapes, as shown in Figure 4. We can find that the optimal classification performance is achieved when the number of typical local surfaces is 30 in SHREC10 and 45 in SHREC15, respectively. We argue that the number of typical local surfaces obtained by unsupervised clustering can largely describe the shape of the entire dataset. Too many typical local surfaces require the Transformer to perform more complex self-attention calculations, that is, more 3D models are required for training.

### 4.4. Robustness on Different Resolutions

The essence of our proposed method is to consider the distribution of different local surface shapes on the model. Compared with MeshCNN based on edge features, our method has a good recognition effect on models of different resolutions. This is due to the use of surface polynomial expressions with few parameters and strong expressive ability. In order to verify the effect of our method under different model resolutions, we simplified the model to different magnitude points for classification experiments.

**Figure 5:**
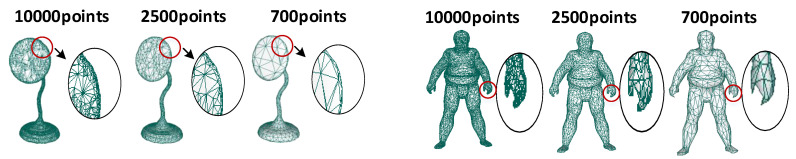
Ablation experiment. The method we propose is based on shape representation, so it still has good results for many simplified models or models with few points. We cited examples of lamp and Sumotori, and showed that their partial shapes were simplified and kept consistent. The first column of the model has 10,000 points, the second column has 2500 points, and the third column has 700 points.

In the experiment, we used the classic quadric error metrics (QEM) algorithm [45] to simplify the mesh model. This mesh simplification method can effectively reduce the number of vertices of the complex mesh while retaining the topological characteristics of the original mesh and can customize the reduced number of vertices. The above two advantages can ensure that our model is simplified to the same vertex order, and the topological shape is kept unchanged, which provides convenience for our comparative experiments. The simplified result is shown in Figure 5.

We reproduce the method in this paper on models of 10,000, 2500, and 700 points, respectively, and the final classification accuracy is shown in Table 4. It is not difficult to find that our method will work no matter whether the resolution is high or low, as long as the surface shape of the model can be roughly unchanged.

### 4.5. Robustness on Different Ratios of Training Set/Test Set

**Figure 6:**
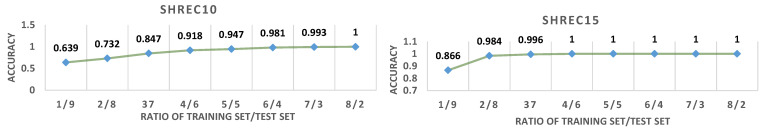
In order to verify that our method can still have good robustness under a small sample data set, we continuously reduce the proportion of the training set and observe its corresponding accuracy. We compare the SHREC10 and SHREC15 data sets when the ratios of the training set to the test set are 1/9, 2/8, 3/7, 4/6, 5/5, 6/4, 7/3, and 8/2.

The current three-dimensional data sets generally have a small sample size, which causes difficulties for network training. For learning with a small number of samples, one solution is data enhancement, and the other is to optimize the network. In the course of the experiment, we found that the method proposed in this paper can obtain features with a large degree of discrimination, and it can still work well when the number of training set samples is small. In order to verify that our method can still have good robustness under a small sample data set, we continuously reduce the proportion of the training set and observe its corresponding accuracy. We compare the SHREC10 and SHREC15 data sets when the ratio of the training set to the test set are 1/9, 2/8, 3/7, 4/6, 5/5, 6/4, 7/3, 8/2. The experimental results are shown in Figure 6.

We can find that for the data set/SHREC10, when the training set/test set is 5/5, the classification accuracy of the network has reached a satisfactory result. Even in SHREC15, when the training set/test set is 3/7, the classification accuracy of the network is almost 100%.

### 4.6. Ablation Experiment of Each Part in The Network Structure

The method proposed in this article mainly includes three parts, namely: 3D ShapeTransformer, ShapesToShapes, ShapesToObject. The first part, ShapeTransformer, obtains the local shape features of the model through a novel mesh Transformer method. The second part, ShapesToShapes, combines the local shapes on the model by designing different multi-head shape attention layers. The last part, ShapesToObject, uses the dynamic routing mechanism to map the shape feature to the entire object. In order to verify the effectiveness of each part, we performed related ablation experiments.

3D Shape Transformer. In this part, we design three sets of ablation experiments: the first one is based on the basic Pointnet, where each point is connected with an MLP to form a latent feature vector; the second is part of our proposed method, which fits the local shape by a polynomial and obtains the latent eigenvectors based on the polynomial parameters; the third one is the method proposed in this paper. On the basis of the second case, a specially designed attention mechanism is added, through which various potential feature vectors can be obtained.

Through the different network inputs designed above, we obtained the model classification experimental results, as shown in Table 5. Taking SHERC10 as an example, the comparison is performed with different front-end inputs under the same experimental architecture. When the input is only 3-dimensional vertex coordinates, the classification accuracy is only 58.7 when having local division and using multi-display parameters as input, the correct rate increases to 89.7 while applying the mesh Transformer proposed in this paper as the front-end input, the correct rate reaches 100. The same trend is observed on the SHREC15 dataset. This proves that our proposed 3D ShapeTransformer part plays a considerable role.

Shapes To Shapes. Inspired by the two-dimensional capsule network, we also hope to combine partial surface shapes into large parts in the three-dimensional model, and finally form the structure of the entire object. In order to solve the problem of combining local surface shapes into larger parts, we conducted experiments and comparisons with or without multi-head shape attention layer in the network.

The experimental comparison results are shown in Table 5. On the dataset SHREC15, the classification accuracy reaches only 23 when we remove the multi-head shape attention layer part, and similarly, on SHREC10, removing the multi-head shape attention layer part makes the classification accuracy drop to 53.2. This is good proof that the multi-head shape attention layer is an indispensable part in our network.

Shapes To Object. The core idea of the dynamic routing algorithm is to increase the weight of the input vector that is highly similar to the output vector. In essence, it can be regarded as a weight distribution process or a clustering process. The higher the similarity, the characteristics of this category will be highlighted. We designed two comparative experiments to prove the importance of ShapesToObject. One is to input the features into the classic PointNet classification network, and the other is to input the capsules containing shape information into the dynamic routing proposed in this paper to perform higher-level feature combination. The experimental results are shown in Table 5.

From the experimental results in the table, it can be seen that the introduction of ShapesToObject has improved the accuracy of 3D model classification regardless of the data set SHREC10 or SHREC15. The classification accuracy was increased from 88.9 and 93.2 to 100, respectively. A large part of the reason is because in PointNet, features are expressed in scalar form, and MLP is introduced to map and combine scalar features. Although this method has good performance on recognition tasks, it loses the most natural features of the 3D model, such as spatial structure information. However, ShapesToObject clusters and maps vector features. Due to the preservation of the spatial structure, the designed neural network can easily achieve excellent classification results.

### 4.7. Time and Space Complexity

Table 6 shows the time and space complexity of our network with several representative methods for classification tasks based on other types of data. Params shows the total number of parameters in the network, and FLOPs shows the number of float operations performed for each input sample, which represent the space and time complexity, respectively.

It can be seen from the comparison that our method has a common problem with the Transformer model, that is, the computational time complexity is high. This is because Transformer needs to calculate the similarity between each input token, so the computational complexity is higher. However, because our method introduces local shape tokens, the parameters of the network are lower than PointNet and MeshNet while still ensuring excellent performance in classification experiments. Since the capsule part of our network adopts dynamic routing for multiple iterations for feature clustering, it is higher in terms of the number of operations, but still within the acceptable range.

## 5. Conclusions

We propose a three-dimensional mesh trans-capsule network combined with vector representation based on the natural interpretable geometric shape information of the model. The network mainly consists of three parts, namely, 3D ShapeTransformer, ShapesToShapes and ShapesToObject. Among them, 3D ShapeTransformer is used to extract the local shape information of the mesh model surface; ShapesToShapes is used to achieve the combination of small surface shapes to large part shapes; ShapesToObject is based on vector-based feature representation and encodes the mapping of parts to a whole. We verify the effectiveness of the vector network proposed in this paper through the classification experiment of the three-dimensional deformation model. Experimental results prove that this method performs well on both SHREC10, SHREC15 and ModelNet40 data sets, and has good generalization and robustness. In future work, we hope to extend the method proposed in this paper to the field of unsupervised learning and realize the generation of 3D mesh models based on local shapes.

## Figures and Tables

**Table 1:** The classification results obtained by different methods under SHREC10.

Methods	Input	Accuracy
Shape-DNA [33]	point	82.67
cShape-DNA [34]	point	78.50
GPS-embedding [35]	point	87.17
BoW [36]	feature	65.94
BoSCCs [36]	feature	85.99
3D MeshConv [37]	mesh	94.37
Our method	mesh	99.95

**Table 2:** The classification results obtained by different methods under SHREC15.

Methods	Input	Accuracy
SVM+HKS [38]	feature	56.9
SVM+WKS [39]	feature	87.5
Shape-DNA [33]	point	64.55
cShape-DNA [34]	point	76.21
GPS-embedding [35]	point	75.13
PointNet [40]	point	69.4
PointNet++ [41]	point	60.2
SpiderCNN [42]	mesh	95.8
3D MeshConv [37]	mesh	97.3
Our method	mesh	99.90

**Table 3:** The classification results obtained by different methods under ModelNet40.

Methods	ModelNet40	Manifold40
PointNet++ [41]	91.7	87.9
PCT [20]	93.2	92.4
SNGC [43]	91.6	-
MeshNet [17]	91.9	88.4
MeshWalker [44]	92.3	90.5
Our method	-	93.7

**Table 4:** Classification accuracy of different resolutions on SHREC10 and SHREC15.

Methods	10,000 points	2500 points	700 points
SHREC10	99.97	99.95	99.95
SHREC15	99.94	99.93	99.90

**Table 5:** Ablation experiment of each part in the network structure.

DataSet	3D ShapeTransformer		ShapesToShapes		ShapesToObject	
SHREC10	Input	Accuracy	Input	Accuracy	Input	Accuracy
	3D points (3 dimensions)	58.7	ShapeTransformer value as primary capsule (without multi-head shape attention layer)	53.2	PointNet	88.9
	Surface parameters (10 dimensions)	89.7			Routing-by-agreement	99.95
	ShapeTransformer value (N dimensions)	99.95	multi-head shape attention layer	99.95	-	-
SHREC15	3D points (3 dimensions)	90.2	ShapeTransformer value as primary capsule (without multi-head shape attention layer)	23.0	PointNet	93.2
	Surface parameters (10 dimensions)	93.8			Routing-by-agreement	99.90
	ShapeTransformer value (N dimensions)	99.90	multi-head shape attention layer	99.90	-	-

**Table 6:** Time and space complexity of classification on the SHEREC15 dataset.

Method	Input	Acc	Params	FLOPs
PointNet [40]	point	69.4	3.5 M	0.44 G
MeshNet [17]	mesh	90.4	4.251 M	0.509 G
MeshCNN [2]	mesh	91.7	1.323 M	0.498 G
Our method	mesh	99.9	2.42 M	0.965 G

## Data Availability

Not applicable.

## References

[1] Hinton G. (1979). Some demonstrations of the effects of structural descriptions in mental imagery. Cogn. Sci..

[2] Hanocka R., Hertz A., Fish N., Giryes R., Fleishman S., Cohen-Or D. (2019). MeshCNN: A Network with an Edge. ACM Trans. Graph..

[3] Baker N., Lu H., Erlikhman G., Kellman P.J., Einhauser W. (2018). Deep convolutional networks do not classify based on global object shape. PLoS Comput. Biol..

[4] Kucker S.C., Samuelson L.K., Perry L.K., Yoshida H., Smith L.B. (2018). Reproducibility and a unifying explanation: Lessons from the shape bias. Infant Behav. Dev..

[5] Dosovitskiy A., Beyer L., Kolesnikov A. (2020). An Image is Worth 16x16 Words: Transformers for Image Recognition at Scale. arXiv.

[6] Kosiorek A., Sabour S., Teh Y.W., Hinton G.E., Wallach H., Larochelle H., Beygelzimer A. (2019). Stacked Capsule Autoencoders. Advances in Neural Information Processing Systems 32.

[7] Zhao Y., Birdal T., Deng H., Tombari F. 3D Point Capsule Networks. Proceedings of the 2019 IEEE/CVF Conference on Computer Vision and Pattern Recognition (CVPR).

[8] Sabour S., Frosst N., Hinton G. (2017). Dynamic Routing between Capsules. Adv. Neural Inf. Process. Syst..

[9] He K., Zhang X., Ren S., Sun J. Deep Residual Learning for Image Recognition. Proceedings of the 2016 IEEE Conference on Computer Vision and Pattern Recognition (CVPR).

[10] Ren S., He K., Girshick R., Sun J. (2017). Faster R-CNN: Towards Real-Time Object Detection with Region Proposal Networks. IEEE Trans. Pattern Anal. Mach. Intell..

[11] He J., Chen J., Liu S., Kortylewski A. (2021). TransFG: A Transformer Architecture for Fine-grained Recognition. arXiv.

[12] Liu Z., Lin Y., Cao Y. (2021). Swin Transformer: Hierarchical Vision Transformer using Shifted Windows. arXiv.

[13] Zhu X., Su W., Lu L., Li B., Wang X., Dai J. (2020). Deformable DETR: Deformable Transformers for End-to-End Object Detection. arXiv.

[14] Carion N., Massa F., Synnaeve G., Usunier N., Kirillov A., Zagoruyko S. (2020). End-to-End Object Detection with Transformers. arXiv.

[15] Hermosilla P., Ritschel T., Vázquez P.P., Vinacua A., Ropinski T. (2018). Monte Carlo Convolution for Learning on Non-Uniformly Sampled Point Clouds. ACM Trans. Graph..

[16] Li Y., Bu R., Sun M., Wu W., Di X., Chen B., Bengio S., Wallach H., Larochelle H., Grauman K., Cesa-Bianchi N., Garnett R. (2018). PointCNN: Convolution On X-Transformed Points. Proceedings of the Advances in Neural Information Processing Systems.

[17] Feng Y., Feng Y., You H., Zhao X., Gao Y. MeshNet: Mesh Neural Network for 3D Shape Representation. Proceedings of the AAAI Conference on Artificial Intelligence.

[18] Biasotti S., Cerri A., Aono M., Hamza A.B. (2016). Retrieval and classification methods for textured 3D models: A comparative study. Vis. Comput..

[19] Rodolà E., Cosmo L., Litany O., Bronstein M.M., Bronstein A.M., Audebert N., Hamza A.B., Boulch A., Castellani U., Do M.N. (2017). Deformable Shape Retrieval with Missing Parts: SHREC’17. Workshop on 3D Object Retrieval.

[20] Guo M., Cai J., Liu Z., Mu T., Martin R.R., Hu S. (2020). PCT: Point Cloud Transformer. arXiv.

[21] Zhao H., Jiang L., Jia J., Torr P., Koltun V. (2020). Point Transformer. arXiv.

[22] Lin K., Wang L., Liu Z. (2021). Mesh Graphormer. arXiv.

[23] Marcos D., Volpi M., Komodakis N., Tuia D. Rotation Equivariant Vector Field Networks. Proceedings of the 2017 IEEE International Conference on Computer Vision (ICCV).

[24] Hinton G.E., Krizhevsky A., Wang S.D. (2011). Transforming Auto-Encoders. Proceedings of the ICANN’11: 21th International Conference on Artificial Neural Networks—Volume Part I.

[25] Srivastava N., Goh H., Salakhutdinov R. (2019). Geometric Capsule Autoencoders for 3D Point Clouds. arXiv.

[26] Lenssen J.E., Fey M., Libuschewski P., Bengio S., Wallach H., Larochelle H., Grauman K., Cesa-Bianchi N., Garnett R. (2018). Group Equivariant Capsule Networks. Advances in Neural Information Processing Systems.

[27] Wang D., Liu Q. An Optimization View on Dynamic Routing between Capsules. Proceedings of the ICLR 2018 Workshop, ICLR 2018.

[28] Hinton G.E., Sabour S., Frosst N. Matrix capsules with EM routing. Proceedings of the 6th International Conference on Learning Representations, ICLR 2018.

[29] Jaiswal A., AbdAlmageed W., Wu Y., Natarajan P., Leal-Taixé L., Roth S. (2019). CapsuleGAN: Generative Adversarial Capsule Network. Proceedings of the Computer Vision—ECCV 2018 Workshops.

[30] Zhao Y., Birdal T., Lenssen J.E., Menegatti E., Guibas L., Tombari F. (2020). Quaternion Equivariant Capsule Networks for 3D Point Clouds. European Conference on Computer Vision.

[31] Wu Z., Song S., Khosla A., Yu F., Zhang L., Tang X., Xiao J. (2015). 3D ShapeNets: A deep representation for volumetric shapes. Proceedings of the 2015 IEEE Conference on Computer Vision and Pattern Recognition (CVPR).

[32] Hu S., Liu Z., Guo M., Cai J., Huang J., Mu T., Martin R.R. (2021). Subdivision-Based Mesh Convolution Networks. arXiv.

[33] Reuter M., Wolter F., Peinecke N. (2006). Laplace-Beltrami spectra as ’Shape-DNA’ of surfaces and solids. Comput.-Aided Des..

[34] Gao Z., Yu Z., Pang X. (2014). A compact shape descriptor for triangular surface meshes. Comput.-Aided Des..

[35] Rustamov R.M. Laplace-Beltrami eigenfunctions for deformation invariant shape representation. Proceedings of the Fifth Eurographics Symposium on Geometry Processing.

[36] Han Z., Liu Z., Vong C.M., Liu Y., Bu S., Han J., Chen C.L.P. (2017). BoSCC: Bag of Spatial Context Correlations for Spatially Enhanced 3D Shape Representation. IEEE Trans. Image Process..

[37] Chen Y., Zhao J., Shi C., Yuan D. (2020). Mesh Convolution: A Novel Feature Extraction Method for 3D Nonrigid Object Classification. IEEE Trans. Multimed..

[38] Bronstein M.M., Kokkinos I. Scale-invariant heat kernel signatures for non-rigid shape recognition. Proceedings of the 2010 IEEE Computer Society Conference on Computer Vision and Pattern Recognition.

[39] Aubry M., Schlickewei U., Cremers D. The wave kernel signature: A quantum mechanical approach to shape analysis. Proceedings of the 2011 IEEE International Conference on Computer Vision Workshops (ICCV Workshops).

[40] Charles R.Q., Su H., Kaichun M., Guibas L.J. PointNet: Deep Learning on Point Sets for 3D Classification and Segmentation. Proceedings of the 2017 IEEE Conference on Computer Vision and Pattern Recognition (CVPR).

[41] Charles R.Q. PointNet++: Deep Hierarchical Feature Learning on Point Sets in a Metric Space. Proceedings of the Neural Information Processing Systems.

[42] Xu Y., Fan T., Xu M., Zeng L., Qiao Y. SpiderCNN: Deep Learning on Point Sets with Parameterized Convolutional Filters. Proceedings of the European Conference on Computer Vision.

[43] Haim N., Segol N., Ben-Hamu H., Maron H., Lipman Y. Surface Networks via General Covers. Proceedings of the 2019 IEEE/CVF International Conference on Computer Vision (ICCV).

[44] Lahav A., Tal A. (2020). MeshWalker: Deep Mesh Understanding by Random Walks. ACM Trans. Graph..

[45] Garland M., Heckbert P.S. Surface simplification using quadric error metrics. Proceedings of the Siggraph.

